# Parasite infection is associated with Kaposi's sarcoma associated herpesvirus (KSHV) in Ugandan women

**DOI:** 10.1186/1750-9378-6-15

**Published:** 2011-09-30

**Authors:** Katie Wakeham, Emily L Webb, Ismail Sebina, Lawrence Muhangi, Wendell Miley, W Thomas Johnson, Juliet Ndibazza, Alison M Elliott, Denise Whitby, Robert Newton

**Affiliations:** 1 Co-infections Studies Programme, Medical Research Council/Uganda Virus Research Institute Uganda Research Unit on AIDS, PO Box 49, Entebbe, Uganda; 2 Epidemiology and Genetics Unit, Department of Health Sciences, Area 3 Seebohm Rowntree Building, University of York, York, YO10 5DD, UK; 3 Department of Infectious Disease Epidemiology, London School of Hygiene and Tropical Medicine, Keppal Street, London, WC1E 7HT, UK; 4 Viral Oncology Section, AIDS and Cancer Virus Program, SAIC_Frederick, National Cancer Institute, PO Box B, Frederick, MD 21702, USA; 5 Department of Clinical Research, London School of Hygiene and Tropical Medicine, Keppal Street, London, WC1E 7HT, UK; 6 Hull York Medical School, University of York, Heslington, YO10 5DD, UK

## Abstract

**Background:**

Immune modulation by parasites may influence susceptibility to bacteria and viruses. We examined the association between current parasite infections, HIV and syphilis (measured in blood or stool samples using standard methods) and antibodies against Kaposi's sarcoma herpesvirus (KSHV), measured by ELISA, in 1915 stored plasma samples from pregnant women in Entebbe, Uganda.

**Results:**

Seroprevalence of KSHV was higher in women with malaria parasitaemia (73% vs 60% p = 0.01), hookworm (67% vs 56% p = 0.001) and *Mansonella perstans *(69% vs 59% p = 0.05); seroprevalence increased with increasing intensity of hookworm infection (p < 0.001[trend]). No associations were found for HIV, five other parasites or active syphilis. These effects were not explained by socioeconomic status or education.

**Conclusions:**

Specific parasite infections are associated with presence of antibodies against KSHV, perhaps mediated via their effect on immune function.

## Background

Infection with KSHV is the underlying cause of Kaposi's sarcoma (KS), although it may not be sufficient [1]. Immune suppression, such as that caused by human immunodeficiency virus (HIV), significantly increases the risk of KS among KSHV infected people and is associated with increased viral load and viral shedding [2-8]. Among people without HIV infection or other forms of overt immune suppression, geographic and temporal variation in the incidence of KS and in the prevalence of KSHV suggest that cofactors may be important in facilitating both transmission and disease [9-18]. Whether cofactors act directly or via effects on the immune system is unclear [19].

Many environmental co-factors for KSHV transmission and disease have been suggested, including volcanic soils [20], limestone [21] and 'oncoweeds' - that is plants with carcinogenic properties or the ability to reactivate KSHV *in vitro *- although epidemiologic evidence of a role for these agents remains scant [22]. Ecological studies in the Mediterranean area found that eradication of mosquitoes and other blood sucking arthropods was associated with declines both in the prevalence of KSHV and in the incidence of KS [9-14,18,23]. The 'promoter arthropod hypothesis' suggests that insect blood feeding increases KSHV transmission through viral reactivation and KS through inflammatory mechanisms associated with the bite [11,14].

Studies of KS in Africa have identified risk factors for KS that might be common to risk of certain parasites, such as exposure to water, high rainfall and walking barefoot [15,24-26]. Previously reported risk factors for KSHV, such as use of surface rather than piped water, may also be consistent with increased exposure to parasites [27]. Ecological associations between malaria and KSHV or KS in Africa are inconclusive [15]. Only one study has attempted to measure parasite burden among cases with KS and controls; KS patients had a higher carriage of certain intestinal helminths than did controls [28].

Parasites impact on immune function [29,30] and could, therefore, modulate the host response to KSHV. The association between EBV (another gamma herpesvirus) and malaria is well documented [31-34]. Parasite-related immune modulation may increase susceptibility to KSHV infection and may also be associated with increased viral shedding and transmission, leading to an increased prevalence of KSHV infection and increased incidence of KS. We tested the hypothesis that parasites may be associated with KSHV by examining associations between current parasite infections and presence of antibodies against KSHV.

## Methods

The investigation was conducted within an existing study in Uganda - the Entebbe Mother and Baby Study (EMaBS) - a large on-going double blind randomised placebo controlled trial designed to determine the impact of helminth infections and their treatment on vaccine responses and infectious disease outcomes. Detailed information about the study design has been reported elsewhere [35]. Briefly, consenting pregnant women resident in Entebbe and Katabi were recruited from the government funded antenatal clinic at Entebbe hospital, Uganda. Blood samples were obtained by venepuncture, and processed for syphilis, HIV serology, CD4 count and for examination for malaria parasites and *Mansonella perstans. *A stool sample was obtained for examination for intestinal helminths. Of note, women were apparently well on the day of enrollment, so infections identified were essentially asymptomatic. Information was collected on clinical and socio-demographic variables and socio-economic status was defined according to a composite variable comprising information on several relevant factors [35].

Maternal plasma samples from the enrolment visit- stored at -80°C at the Uganda Virus Research Institute (UVRI), Entebbe, Uganda - were identified for 1915 women. KSHV serologic testing was based on ELISA for recombinant proteins to K8.1, a KSHV structural glycoprotein expressed during lytic infection, and for ORF 73, a nuclear antigen expressed during latency as previously described [36,37]. Each ELISA plate contained three positive and three negative controls for quality control and cut-off calculation. Both K8.1 and ORF 73 assay have high performance accuracy with a sensitivity of 98.78% and 89.02% respectively and specificity of 98.79% and 97.57% respectively [36]. The ELISAs were performed at the Uganda Virus Research Institute (UVRI) by the study lead and a technician, both of whom were blinded to patient details. The two assays were used to define evidence of KSHV - individuals were considered to be seropositive if they were positive to either assay and negative if both ELISA assays were negative. The assays were transferred to UVRI from the Viral Oncology Section (VOS), National Cancer Institute (NCI), USA and analysis of the positive and negative controls showed comparable performance at NCI and UVRI. The geometric mean optical density (OD) of the ORF 73 positive and negative controls was 2.50 and 0.04 respectively at VOS, NCI and 2.62 and 0.06 respectively at UVRI. The K8.1 positive and negative controls were 2.23 and 0.10 at NCI and 2.22 and 0.06 at UVRI. Analysis of 375 samples tested in duplicate at both VOS, NCI and UVRI resulted in Kappa values of 0.89 for the K8.1 ELISA and 0.86 for the ORF 73 ELISA.

For parasite intensities, hookworm was measured by egg counts in stool and catogorised as light (< 1,000 eggs per gram (epg) of stool), moderate (1,000 to 3,999 epg) and heavy infection (≥4,000 epg) [35,38]. The intensity of malaria infection was categorised as being below ("low") or above ("high") the median parasite count, per 200 white blood cells. For microfilariae, tertiles of the number of filaria per millilitre of blood were used to categorise into low, medium and high infection intensity.

Data were analysed using Stata11SE (StataCorp LP, College Station, Texas, USA). Potential associations between KSHV seropositivity and each potential risk factor were estimated using the Pearson chi-squared test or Fisher's exact test where expected numbers were small. Odds ratios (ORs) and 95% confidence intervals (CIs) were calculated using logistic regression modeling controlling for age and other possible confounders. The possibility of multiple parasite infections increasing risk of KSHV seropositivity more or less than expected under a multiplicative model was assessed by fitting terms for interactions between parasites in the logistic regression model. The agreement between K8.1 and ORF73 ELISA assays was assessed formally by calculating a Kappa statistic. All p values were 2-sided and we considered p<0.05 to be statistically significant.

## Results and Discussion

The median age of women in the study was 23 years (IQR 19-27); most were in the third trimester of pregnancy (54% (1032/1915) with 46% (879/1915) in the second). The seroprevalence of HIV was 10% (193/1915) and the median CD4 count among those who were seropositive was 551 (IQR 368-796). The highest level of educational attainment reached by the majority of women was primary (50% [961/1911]), with 62% (1191/1915) describing themselves as unemployed or housewives and 82% (1561/1905) reporting a personal income of less than 30,000 Ugandan Shillings (approx. $12USD) per month. The prevalence of antibodies to K8.1 was 41% and to ORF 73 was 52%. 32% of women were seropositive to both antigens and 61% had antibodies to either ORF 73 and/or K8.1. There was moderate concordance between latent KSHV ORF 73 and lytic KSHV K8.1 assays in detecting KSHV seropositivity (k = 0.43), consistent with previous studies [37,39]. Prevalence of antibodies did not change significantly with age, although the age range of study participants was relatively narrow. Previous studies of women in a similarly narrow age range in Africa have showed little or no association with age [27,37,39,40]. As expected, prevalence decreased with increasing maternal education and household socioeconomic status [27]

Common infections among the participants were hookworm (44%), *Mansonella perstans *(21%), *Schistosoma mansoni *(18%), asymptomatic *Plasmodium falciparum *parasitaemia (10%) and HIV (10%). The prevalence of antibodies to K8.1, ORF 73, both antigens and either antigen was 41%, 52%, 32% and 61% respectively. Unadjusted ORs for the association of infections (including eight current parasitic infections), socio-demographic and behavioral factors with antibodies against KSHV are shown in Table 1. In Table 2, the variables found to be associated with KSHV serostatus have been examined again with adjustment for each other. Seropositivity to KSHV was significantly associated with malaria parasitaemia, hookworm and *Mansonella perstans*. The prevalence of antibodies to KSHV increased with increasing intensity of hookworm infection (p < 0.001 [trend]; as measured by egg counts in stool), from 56% among those with no infection to 67% in those with light/moderate infection (12 - the limit of detection - to 3,999 eggs per gram (epg) of stool) to 72% in those with heavy infection (≥4,000 epg); no consistent trends were observed for malaria parasite density or *Mansonella perstans *intensity, but most infections were light (Table 3).

**Table 1:** Crude associations with antibodies against KSHV and sociodemographic and clinical risk factors among Ugandan women.

Factor	Prevalence of women KSHV seropositive*	OR (95% CI)	P**
**Age**			
14-19 years	65% (288/443)	1	
20-24 years	62% (446/717)	0.89 (0.70-1.14)	
25-29 years	56% (252/444)	0.71(0.54-0.93)	
30-34 years	58% (125/216)	0.74 (0.53-1.03)	
> = 35 years	55% (52/94)	0.67 (0.43-1.05)	0.007 [trend]
**Maternal education**			
None	74% (50/68)	1	
Primary	64% (617/960)	0.65 (0.37-1.12)	
Senior	59% (418/713)	0.51 (0.29-0.89)	
Tertiary	45% (76/169)	0.29 (0.16-0.55)	<0.001 (trend)
Household SES***			
1 (lowest)	69% (75/109)	1	
2	71% (112/158)	1.10 (0.65-1.88)	
3	64% 369/581)	0.79 (0.51-1.22)	
4	58% 310/531)	0.64 (0.41-0.99)	
5	57% (222/389)	0.60 (0.38-0.95)	
6 (highest)	50% (55/111)	0.45 (0.26-0.77)	<0.001 [trend]
**HIV**			
Negative	60% (1033/1721)	1	
Positive	67% (130/193)	1.38 (1.00-1.89)	0.04
**Malaria parasites**			
No	60% (1010/1697)	1	
Yes	73% (135/185)	1.84 (1.31-2.58)	<0.001
**Active syphilis**			
No	61% 1110/1833)	1	
Yes	68% (52/76)	1.41 (0.86-2.31)	0.25
**Hookworm**			
No	56% (594/1070)	1	
Yes	67% (562/836)	1.64 (1.36-1.98)	<0.001
***Mansonella perstans***			
No	59% (884/1508)	1	
Yes	69% (277/402)	1.55 (1.23-1.96)	<0.001
***Schistosoma mansoni***			
No	61% (952/1568)	1	
Yes	60% (204/338)	1.0 (0.78-1.25)	0.90
***Strongyloides stercoralis***			
No	61% (1007/1665)	1	
Yes	63% (144/230)	1.10 (0.82-1.46)	0.54
***Trichuris trichiura***			
No	60% (1044/1739)	1	
Yes	67% (112/167)	1.36 (0.97-1.90)	0.07
***Ascaris lumbricoides***			
No	61% (1125/1859)	1	
Yes	66% (31/47)	1.27 (0.69-2.33)	0.45
***Trichostrongylus *species**			
No	61% (1149/1887)	1	
Yes	37% (7/19)	0.38 (0.15-0.96)	0.03
**Use of mosquito spray in the home**			
No	62% (942/1510)	1	
Yes	55% (219/400)	0.73(0.58-0.90)	0.005
**Use of bed net**			
No	61% (574/952)	1	
Yes	60% (587/959)	1.04 (0.87-1.25)	0.68
**Walk barefoot**			
Yes	61% (925/1505)	1	
No	58% (236/407)	1.15 (0.92-1.44)	0.20

* Individuals were considered KSHV positive if they had a positive ORF 73 and/or K8.1 ELISA. Participants were considered negative if both ELISAs were negative.

** All estimated using Chi-squared test except for *Trichostrongylus *species which was estimated using a Fishers exact test. All tests of statistical significance two sided.

*** Socio-economic status

**Table 2:** Adjusted associations with antibodies against KSHV and sociodemographic and clinical risk factors among Ugandan women.

	KSHV seropositive*	
Factor**	**OR (95% CI)**	P***

**Age**	1	
Trend OR	0.94 (0.86-1.02)	0.14
		
**Maternal education**	1	
Trend OR	0.79 (0.69-0.91)	0.001
		
Household SES****	1	
Trend OR	0.90 (0.83-0.98)	0.01
		
**HIV status**		
Negative	1	
Positive	1.35 (0.97-1.89)	0.08
		
**Malaria parasites**		
No	1	
Yes	1.60 (1.12-2.27)	0.01
		
**Hookworm**		
No	1	
Yes	1.40 (1.14-1.71)	0.001
		
***Mansonella perstans***		
No	1	
Yes	1.29 (1.00-1.65)	0.05

* Individuals were considered KSHV positive if they had a positive ORF 73 and/or K8.1 ELISA. Participants were considered negative if both ELISAs were negative,

**All factors are adjusted for each other

***All tests of statistical significance two sided.

**** Socio-economic status

**Table 3:** Crude associations with antibodies against KSHV and intensity of parasite infection in Uganda women.

Infection	Prevalence of women KSHV seropositive*	OR (95% CI)	P**
**Hookworm**			
Uninfected	56% (594/1070)	1	
Light	67% (478/714)	1.62 (1.33-1.98)	
Moderate	67%(61/91)	1.62 (1.04-2.56)	
Heavy	72% (23/32)	2.05 (0.94-4.47)	<0.001[trend]
**Malaria parasites**			
No	40% (687/1697)	1	
Low	75% (68/91)	2.01 (1.24-3.26)	
High	66% (81/123)	1.31 (0.89-1.93)	0.02[trend]
***Mansonella perstans***			
Uninfected	59% (884/1508)	1	
Light	69% (98/142)	1.57 (1.09-2.28)	
Moderate	74% (68/92)	2.00 (1.24-3.22)	
Heavy	66% (111/169)	1.35 (0.97-1.89)	0.02[trend]

* Individuals were considered KSHV positive if they had a positive ORF 73 and/or K8.1 ELISA. Participants were considered negative if both ELISAs were negative.

** All estimated using Chi-squared test. All tests of statistical significance two sided.

Mode of KSHV transmission is yet to be fully elucidated, but high acquisition rates during childhood imply a non-sexual route [7,27,37,40-49]. In studies of mother-child pairs [37,40,50], the impact of HIV on KSHV seropositivity is unclear with some studies reporting a positive impact [37] and others reporting borderline or null association [40,50]. We observed no statistically significant association between HIV and KSHV seropositivity. There was no association between KSHV seropositivity and CD4 count in HIV infected women (p = 0.13). The lack of association with syphilis is consistent with previous studies reporting no association with KSHV and markers of sexual behavior [37,47,51,52]. In unadjusted analyses, the use of insecticide in the home was associated with a lower prevalence of antibodies against KSHV (p = 0.005) although use of a bed net and walking barefoot (a risk factor for hookworm infection) was not. Effects of increasing numbers of infections on KSHV seropositivity combined multiplicatively; there were no interactions between the effects of HIV, malaria parasitaemia, hookworm or *Mansonella perstans *on KSHV infection (results not shown). KSHV seropositivity was not associated with trimester or pregnancy duration as measured in months. The results for ORF 73 and K8.1 separately, did not materially differ.

This study has a number of important limitations. It is possible that associations arose as a result of residual confounding by socio-economic status (SES), although adjustment for certain markers of SES had no effect on the findings. Furthermore, the work was cross sectional and so associations identified should be confirmed in longitudinal studies. Also, the study participants were pregnant and pregnancy itself may modulate immune function. However, since all comparisons were internal within the study (i.e. comparing one group of pregnant women with another), it is difficult to see how this could have impacted on the results.

## Conclusions

The findings reported here provide evidence of an association between specific parasites and presence of antibodies against KSHV. Specific parasite infections may increase KSHV replication or cause reactivation, thereby increasing the likelihood of detecting antibodies against KSHV. Alternatively, specific parasites may increase susceptibility to infection - we cannot, in this study, distinguish between these two possibilities. Co-factors for KSHV transmission and disease have been sought to explain the elevated prevalence of KSHV and incidence of KS in sub-Saharan Africa. Data presented here suggest that parasites may constitute one such co-factor. Further epidemiological and laboratory studies are needed to fully understand the role of parasites as a risk factor for infection with KSHV.

### Ethical approval

Ethical approval for this study was obtained from three bodies: Uganda Virus Research Institute Science and Ethics Committee, Entebbe, Uganda; Uganda National Council for Science and Technology; and the University of York, UK.

## Competing interests

The authors declare that they have no competing interests.

## Authors' contributions

KW conceived and coordinated the study, carried out the KSHV ELISA assays, performed the statistical analysis and drafted the manuscript, ELW performed the statistical analysis and helped to draft the manuscript, IS carried out KSHV ELISA assays, LM managed the study database, WM set up, validated and carried out the KSHV ELISA assays and helped to draft the manuscript, WTM helped with statistical analysis and drafting the manuscript, JN was project leader for the EMaBS cohort, AME is principle investigator for the EMaBS cohort, conceived the study and helped with statistical analysis and drafting the manuscript, DW is head of VOS, NCI, conceived the study and drafted the manuscript and RN drafted the manuscript. All authors have read and approved the final manuscript.
